# Epidemiology of second trimester induced abortion in Ethiopia: a systematic review and meta-analysis

**DOI:** 10.3389/fgwh.2025.1452114

**Published:** 2025-04-28

**Authors:** Mesfin Abebe, Tsion Mulat Tebeje, Nebiha Yimer, Tesfaye Temesgen, Getnet Melaku, Habtamu Endashaw Hareru

**Affiliations:** ^1^ Department of Midwifery, College of Health Sciences and Medicine, Dilla University, Dilla, Ethiopia; ^2^ School of Public Health, College of Health Sciences and Medicine, Dilla University, Dilla, Ethiopia

**Keywords:** second trimester induced abortion, systematic review, meta-analysis, epidemiology, Ethiopia

## Abstract

**Background:**

Second-trimester induced abortion refers to the termination of a pregnancy occurring between 13 and 28 weeks of gestation in Africa. These abortions are particularly concerning due to the heightened risk of complications and maternal mortality. In Ethiopia, there is a lack of nationally representative data regarding the magnitude and associated factors of second-trimester induced abortions. This systematic review and meta-analysis aimed to ascertain the pooled magnitude and identify the factors associated with second-trimester induced abortions in Ethiopia.

**Methods:**

The PRISMA guidelines were used to review and report this study. A systematic literature search was conducted to identify relevant articles from online databases, including PubMed/MEDLINE, Web of Science, Google Scholar, and Ethiopian University online repositories. Data were extracted using an Excel data extraction format, and analysis was performed using Stata version 17. A meta-analysis was conducted using a random-effects model, and subgroup analysis was performed based on the year of publication and sample size to identify the source of heterogeneity. To determine publication bias, a funnel plot, and Egger's regression test were conducted.

**Results:**

In this review, a total of ten articles encompassing 4,466 participants were analyzed. The pooled prevalence of second-trimester induced abortion in Ethiopia was found to be 29.10% (95% CI: 19.96–38.24; *I*² = 97.84, *P* < 0.000). Delay in confirming pregnancy (POR = 4.33, 95% CI: 2.25, 8.32), nature of the menstrual cycle (POR = 0.32, 95% CI: 0.18, 0.56), residence (POR = 0.38, 95% CI: 0.30, 0.49), and pregnancy intention (POR = 0.28, 95% CI: 0.18, 0.42) were significantly associated with second-trimester induced abortion.

**Conclusions:**

The magnitude of induced second-trimester abortions in Ethiopia is significantly higher than global data. This meta-analysis identifies factors associated with second-trimester abortions, including delayed pregnancy confirmation, irregular menstrual cycles, rural residency, and unplanned pregnancies. The findings highlight the urgent need for targeted interventions to address these factors and decrease the incidence of second-trimester abortions. Encouraging early pregnancy testing and confirmation to reduce delays, raising awareness about the importance of regular menstrual cycles and seeking medical advice for irregularities, improving healthcare services in rural areas to reduce disparities, and strengthening family planning and counseling services can help mitigate unplanned pregnancies and induced abortions.

**Systematic Review Registration:**

PROSPERO (CRD42022383559).

## Background

Induced abortion is defined as the deliberate termination of a pregnancy before 28 weeks of gestation or when the fetus weighs less than 1,000 g and cannot live independently outside of the womb (1). While the World Health Organization (WHO) recognizes comprehensive abortion care as an essential health service, many countries only allow abortion under certain conditions and around 20 countries provide no legal grounds for it at all. Additionally, over three-quarters of countries impose legal penalties for abortion (2). Every year, there are an estimated 73.3 million abortions globally, with a rate of 39 per 1,000 women aged 15–49. Of these, approximately 25 million are unsafe, and 97% of unsafe abortions occur in developing countries (3). These abortions make up 61% of unintended pregnancies and 29% of all pregnancies (2). Annual abortion incidence decreased worldwide in the 1990s and early decades of the twenty-first century, but this decline was driven by high-resource settings, while abortion rates in low- and middle-resource countries remained stable (4, 5). Over the 30 years, the global proportion of unintended pregnancies that resulted in abortion increased from 51% to 61% in 2015–19 (5). It's crucial to recognize that these estimates are based on limited data and controversial methodologies. Researchers face challenges in measuring abortion incidence due to the difficulty in assessing its scale, especially in environments where the procedure is conducted secretly, either due to legal restrictions or lack of trained, licensed practitioners (6–8). As part of a broader strategy to improve maternal health outcomes, it is recommended that states should help women avoid abortion where possible. According to the ICPD Program of Action, governments should help women avoid abortion and ensure that abortion is not used as a method of family planning (9, 10).

Second-trimester induced abortion is the termination of a pregnancy between the gestational age of 13 and 28 weeks in Africa (11, 12). Although it is less common than first trimester abortion, it does occur in both developed and developing countries (13). It remains a public health concern because it leads to higher rates of maternal morbidity and mortality compared to first trimester abortions, particularly in low-resource countries where access to safe second-trimester abortions is limited (11). Although unsafe abortions are largely responsible for complications, even ’safe’ abortions can result in significant problems if emergency services are inadequate. Even though second-trimester abortion only accounts for a small percentage of all induced abortions, it is associated with two-thirds of major abortion-related complications and more than half of abortion-related mortality (14).

Globally, about 7.9% of maternal deaths are attributed to all types of abortion, including spontaneous abortion (i.e., miscarriage), induced abortion, and ectopic pregnancies (15). Many of these deaths are caused by unsafe abortion, and a significant number of them occur in the second trimester (4, 11, 12). So unsafe abortion is a major contributor to death and serious health issues among women in developing countries (16).

Obtaining accurate data on the global incidence of second-trimester abortions is challenging, primarily because many countries with legal restrictions on abortion do not collect or report these statistics (17, 18). However, it is estimated that around 10%–15% of all induced abortions occur during the second trimester, contributing significantly to abortion-related complications and deaths (17). In sub-Saharan Africa, two-thirds of complications from unsafe abortions happen during the second trimester (19). Many abortions in sub-Saharan Africa, including Ethiopia, still lead to preventable health consequences for women (20). Abortion during the second trimester exposes women to a higher risk of medical complications, and a higher risk of mortality and morbidity than abortion during the first trimester (21, 22).

A study in Nepal found a higher proportion of second-trimester abortions between the ages of 21 and 25 (23). Several observational studies on the magnitude of Second trimester-induced abortion and its associated factors have been conducted in Ethiopia. According to these various studies, the magnitude of second-trimester induced abortion among pregnant women seeking abortion care in Ethiopia ranged from 12.06% to 58.0% (12, 19, 24–28). Individual studies revealed significant variation in the magnitude of second-trimester induced abortion as well as inconsistencies in its associated factors in different areas of Ethiopia (12, 25, 27, 29, 30). As a result, it is still unknown why there is variation in the prevalence of second-trimester induced abortion and associated factors among women in Ethiopia. The pooled magnitude of induced second-trimester abortion in Ethiopia is also unknown, and this is another gap in the literature. Therefore, the purpose of this systematic review and meta-analysis is to assess the magnitude and associated factors of second-trimester induced abortion in Ethiopia.

## Methods

This systematic review and meta-analysis adhered to the Preferred Reporting Items for Systematic Review and Meta-Analysis (PRISMA 20) (31) (Supplementary File S1). This systematic review and meta-analysis protocol was registered in the International Prospective Register of Systematic Reviews (PROSPERO) with reference number CRD42022383559.

The review question was developed using the CoCoPop framework, tailored for prevalence studies. The structure is as follows:

**Condition:** Second trimester induced abortion.
**Context:** Ethiopia.
**Population**: Pregnant women who have undergone induced abortion services.

### Eligibility criteria

The observational studies, both published and unpublished, conducted in Ethiopia on the magnitude and associated factors of second-trimester induced abortion were included. The review includes English-language case-control, cross-sectional, and cohort studies of any sample size published between January 2010 and November 2024. Articles with incomplete information after repeated requests from the corresponding authors, as well as reports, reviews, and those fully inaccessible, were excluded from the study.

### Outcomes

The primary outcome was the prevalence of second-trimester induced abortion and the secondary outcome was factors associated with second-trimester induced abortion.

#### Searching strategy

To confirm the absence of similar studies in Ethiopia, the Database of Abstracts of Reviews of Effects (DARE) and the Cochrane Database of Systematic Reviews (CDSR) were initially searched. A systematic literature search was done to identify relevant articles from online databases PubMed MEDLINE, Embase, Web of Science, Google Scholar, and Ethiopian University online repositories. The search strategies in PubMed for the MeSH terms and text words were (magnitude) OR (prevalence)) AND (second trimester induced abortion)) AND (associated factors)) AND (Ethiopia). (“magnitude"[All Fields] OR “magnitudes"[All Fields] OR (“epidemiology"[MeSH Subheading] OR “epidemiology"[All Fields] OR “prevalence"[All Fields] OR “prevalence"[MeSH Terms] OR “prevalance"[All Fields] OR “prevalences"[All Fields] OR “prevalence s"[All Fields] OR “prevalent"[All Fields] OR “prevalently"[All Fields] OR “prevalents"[All Fields])) AND ((“pregnancy trimester, second"[MeSH Terms] OR (“pregnancy"[All Fields] AND “trimester"[All Fields] AND “second"[All Fields]) OR “second pregnancy trimester"[All Fields] OR (“second"[All Fields] AND “trimester"[All Fields]) OR “second trimester"[All Fields]) AND (“abortion, induced"[MeSH Terms] OR (“abortion"[All Fields] AND “induced"[All Fields]) OR “induced abortion"[All Fields] OR (“induced"[All Fields] AND “abortion"[All Fields]))) AND (“ethiopia"[MeSH Terms] OR “ethiopia"[All Fields] OR “Ethiopia"[All Fields]).

### Quality assessment

The titles and abstracts of studies to be included in this systematic review and meta-analysis were reviewed independently by two authors, and articles were exported to Endnote 20 to manage duplications. Disagreements on study inclusion and exclusion were resolved through the involvement of the third author. The modified version of the Newcastle-Ottawa Quality Assessment Scale was used to evaluate the quality of studies (32, 33). It comprises three components. The initial component evaluates the methodology of the study and is scored out of five stars. The second component assesses comparability and is scored out of three stars. The third component evaluates the statistical analysis and outcome of each study and is scored out of two points. Finally, high-quality articles with at least 7 points out of 10 possible points for cross-sectional studies and case-control studies were included in this systematic review and meta-analysis (34) (Supplementary File S2).

### Data extraction

A Microsoft Excel worksheet was used to prepare the data extraction format. Two authors prepared the data extraction format for extracting data on the magnitude and associated factors of second-trimester induced abortions. The data extraction format for the primary outcome included the first author, region (according to Ethiopian political administration), study period, publication year, study design, sample size, response rate, and prevalence of second-trimester induced abortion. The data extraction format was also prepared for each specific factor (delay to confirm pregnancy, nature of menses, residence, and pregnancy intention) for the second outcome (associated factors). During the data extraction, any disagreements between the two authors were discussed and resolved by the third author.

### Data processing and analysis

After extracting all relevant data, the data were exported from Excel into STATA 17 for analysis. A pooled prevalence with a 95% confidence interval was calculated. The results of the meta-analyses were displayed in forest plots and tables. The overall pooled magnitude of second-trimester induced abortion and its associated factors were estimated using a random-effect model. The statistical significance level was declared at a *p*-value less than 0.05. The *I*^2^ statistic test was used to examine heterogeneity (35). *I*^2^ values of 25%, 50%, and 75%, respectively, represented low, moderate, and large heterogeneity. To examine sources of heterogeneity, subgroup analysis, and meta-regression were undertaken based on publication year, response rate, and sample size. Visual inspection of funnel plots (36) and Egger's test (37) were used to investigate publication bias. A *p*-value less than 0.05 on Egger's test confirmed statistically significant publication bias. Finally, the result was presented in text, figures, and table format.

## Results

### Search results

**Figure 1:**
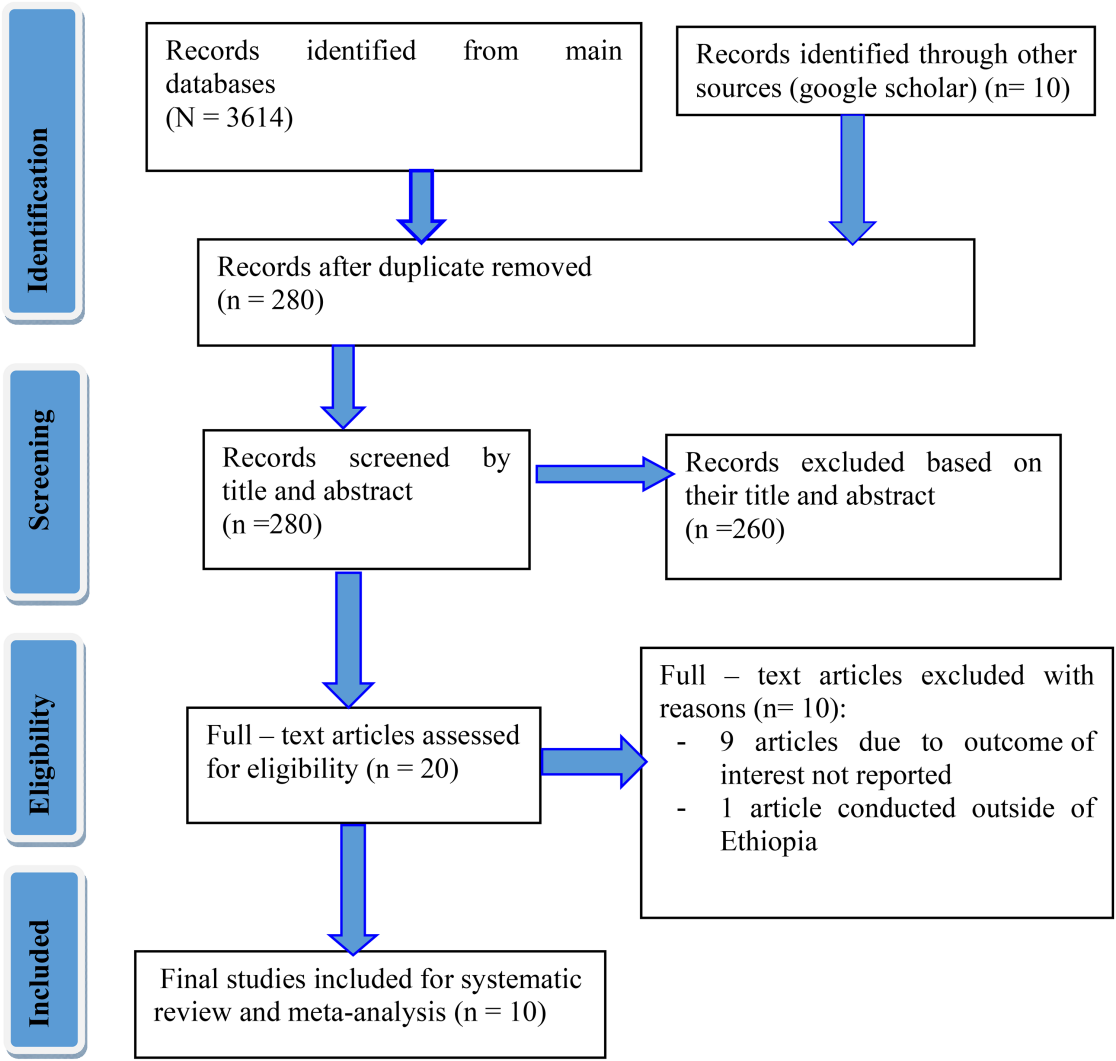
PRISMA flow diagram of the study.

A total of 3,624 studies were retrieved from various databases and other electronic resources. All collected studies were transferred to Endnote version 20 reference manager for screening, and 3,334 studies were eliminated owing to duplication. Again, 260 studies were eliminated after reading their title and abstracts. Twenty (20) articles were evaluated for their eligibility requirements, and finally, 10 studies that met the inclusion criteria were included (Figure 1).

### Characteristics of included studies

This meta-analysis comprised a total of ten studies, including nine that were published and one unpublished, involving 4,466 participants. Out of these studies, eight were cross-sectional studies (12, 19, 24–28, 38), while two were case-control studies (29, 30). In terms of geographical distribution, four regions and one administrative city were represented: Oromia region, South Ethiopia region, Amhara region, Harar region, and Addis Ababa. In the included primary studies, response rates ranged from 88.5% in Jimma, Oromia (24) to 100% in studies conducted in Harar, Oromia, and Amhara (19, 29, 38). Nine articles (12, 19, 24–30, 38) from the included articles were published and made available in the major databases, and one study (38) was located online in the form of a preprint. The included studies’ quality was evaluated using the NOS quality assessment instrument. Based on the NOS quality assessment scores, studies with quality scores greater than 7 were regarded as having a low risk for bias (Supplementary File S2). Abdi T et al. (19) reported a higher proportion of induced abortions occurring during the second trimester, followed by Kebede K et al. (26). In contrast, Bonnen I et al. (24) found a lower proportion (see Table 1).

**Table 1:** Characteristics of included studies in this systematic review and meta-analysis on the magnitude and associated factors of second-trimester induced abortion in Ethiopia, 2024.

Author	Publication year	Study area	Study design	Study setting	Sample size	Response rate	Magnitude
Tesfaye et al. (25)	2020	Amhara	Cross-sectional	Facility based	262	94.3	29.6%
Mohammed et al. (38)	2021	Harar	Cross-sectional	Facility based	835	100	18.2%
Abebe et al. (12)	2022	South Ethiopia	Cross-sectional	Facility based	353	97.3	23.0%
Kebede et al. (26)	2020	Addis Ababa	Cross sectional	Facility based	246	96.7	53.4%
Mulat et al. (27)	2015	Amhara	Cross-sectional	Facility based	422	98.6	19.2%
Bonnen et al. (24)	2014	Oromia	Cross-sectional	Facility based	936	88.5	12.06%
Abdi et al. (19)	2024	Oromia	Cross-sectional	Facility based	260	100	58.0%
Dagnaw et al. (28)	2024	Amhara	Cross-sectional	Facility based	405	94.07	21.5%
Wasihun et al (30)	2021	Amhara	Case control	Facility based	357	97.2	–
Addisu et al. (29)	2024	Amhara	Case-control	Facility based	390	100	–

### Magnitude of second-trimester induced abortion

**Figure 2:**
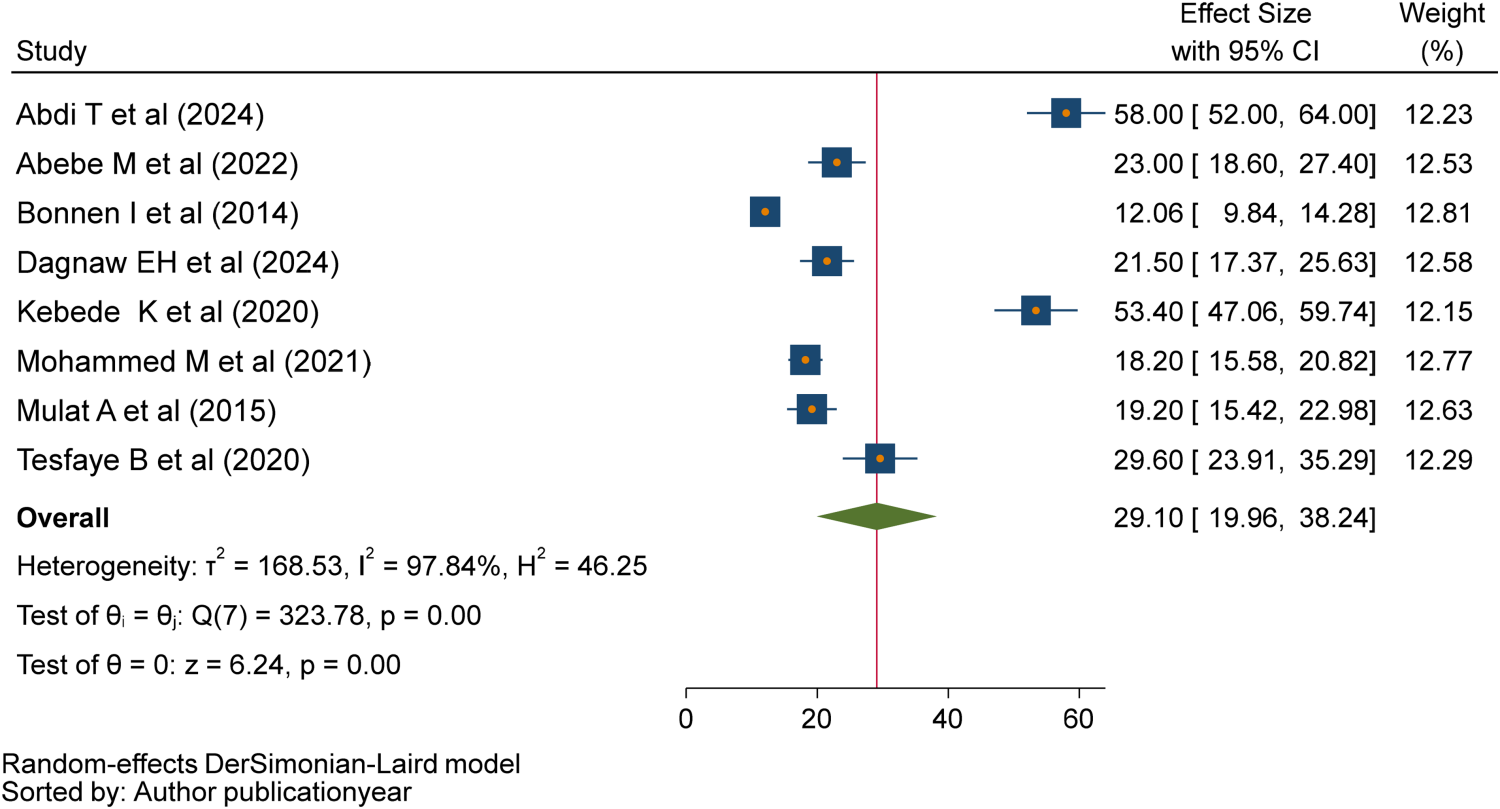
Forest plot of pooled magnitude of second-trimester induced abortion in Ethiopia, 2024.

Eight primary studies (12, 19, 24–28, 38) were included in this meta-analysis to determine the pooled magnitude of second-trimester induced abortion in Ethiopia. The pooled magnitude of induced abortion in the second trimester was 29.10% (95% CI: 19.96–38.24; *I*^2^ = 97.84, *P* < 0.001). The pooled magnitude was determined using a random effect model (Figure 2). Because they were case-control studies, two studies (29, 30) were excluded from the estimation of the pooled magnitude of second-trimester induced abortion.

### Subgroup analysis

**Figure 3:**
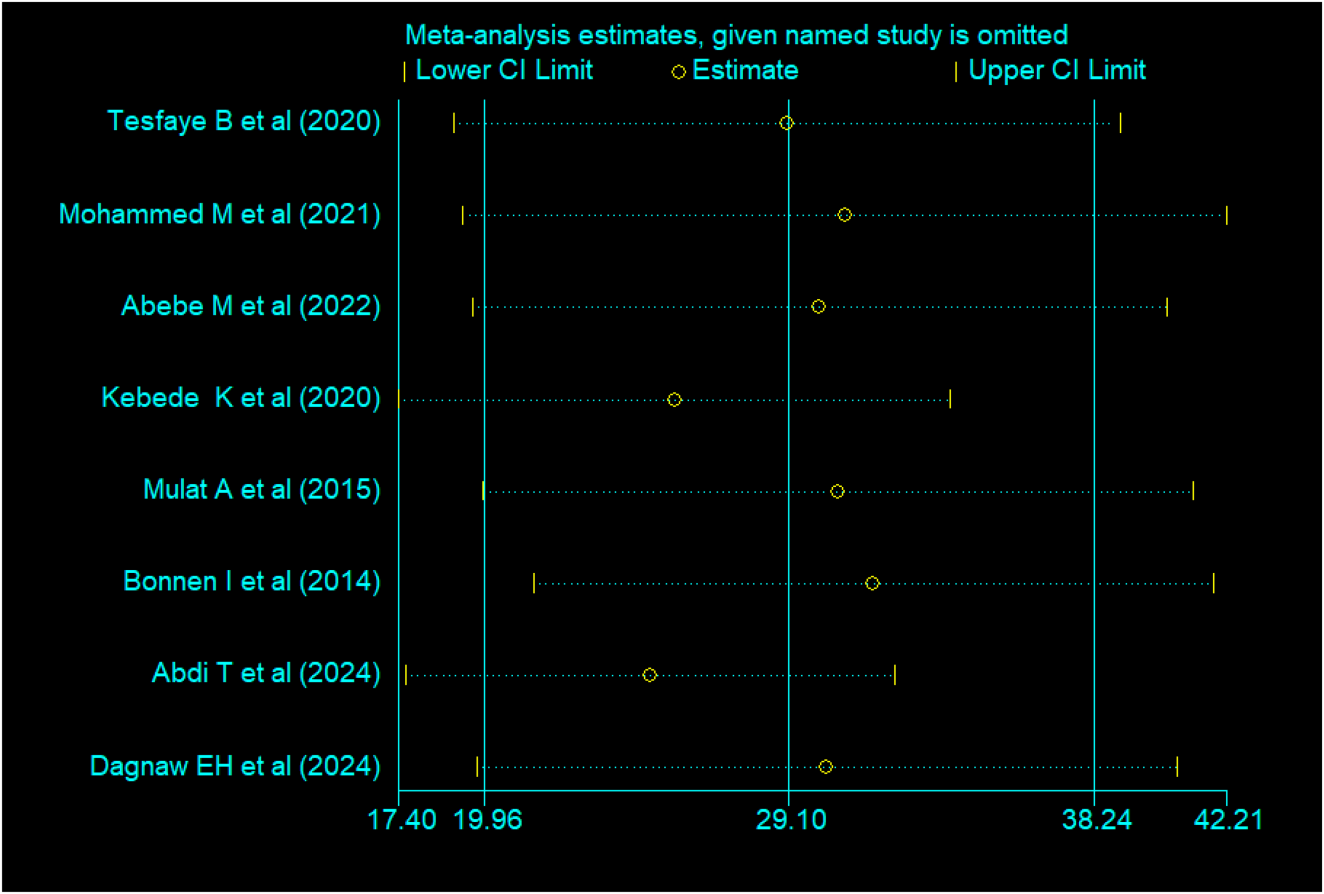
Sensitivity analysis for the magnitude of second-trimester induced abortion in Ethiopia, 2024.

To identify the source of heterogeneity and reduce discrepancies between primary study estimates, subgroup analysis was conducted based on sample size and year of publication. Results showed that studies published in 2020 or later had a higher magnitude of second-trimester induced abortion (*P* = 33.37%; CI: 21.60–45.94) compared to those published before 2020 (*P* = 15.46%; CI: 8.47–22.45) (Figure 3). Studies with sample sizes smaller than 464 (mean) showed a higher pooled rate of second-trimester induced abortions (*P* = 33.96%; CI: 21.68–46.24) compared to studies with sample sizes of 464 or more (*P* = 15.09%; CI:9.07–21.11) (Table 2). Sensitivity analysis and meta-regression were also conducted, in addition to subgroup analysis, to identify the source of heterogeneity.

**Table 2:** Sub-group analysis of the pooled magnitude of second-trimester induced abortion in Ethiopia based on publication year and sample size.

Variables	Characteristics	Pooled magnitude (95%CI)	*I*^2^ (*p*-value)
Publication year	<2020	15.46% (8.47–22.45)	90.18% (<0.001)
	≥2020	33.37% (21.60–45.94)	97.76% (<0.001)
Sample size (mean)	<464 (mean)	33.96% (21.68–46.24)	97.40% (<0.001)
	≥464 (mean)	15.09% (9.07–21.11)	91.88% (<0.001)

### Sensitivity analysis

Sensitivity analyses were performed for effect sizes of all of the studies on second trimester induced abortion to identify the possible source of heterogeneity and to find out the effect of one study on the overall estimate. However, none of the studies were found to show a statistically significant source of heterogeneity nor a significant influence in all the analyses (Figure 3).

### Meta-regression

In addition to conducting subgroup analysis based on sample size, response rate, and year of publication, a meta-regression was performed using these factors as covariates to identify the source of heterogeneity at a 5% significance level. However, the results of the meta-regression showed that there was no statistical association between the pooled magnitude of second-trimester induced abortion and either the year of publication or sample size (Table 3).

**Table 3:** Meta-regression analysis of factors affecting study heterogeneity.

Source of heterogeneity	Coefficient	Standard error	Z	*P*>|z|	95% confidence interval
Year of publication	0.59	1.86	0.32	0.749	−3.06 to 4.25
Sample size	−0.03	0.02	−1.40	0.162	−0.08 to 0.01
Response rate	0.65	1.70	0.38	0.702	−2.69 to 3.99

### Publication bias

**Figure 4:**
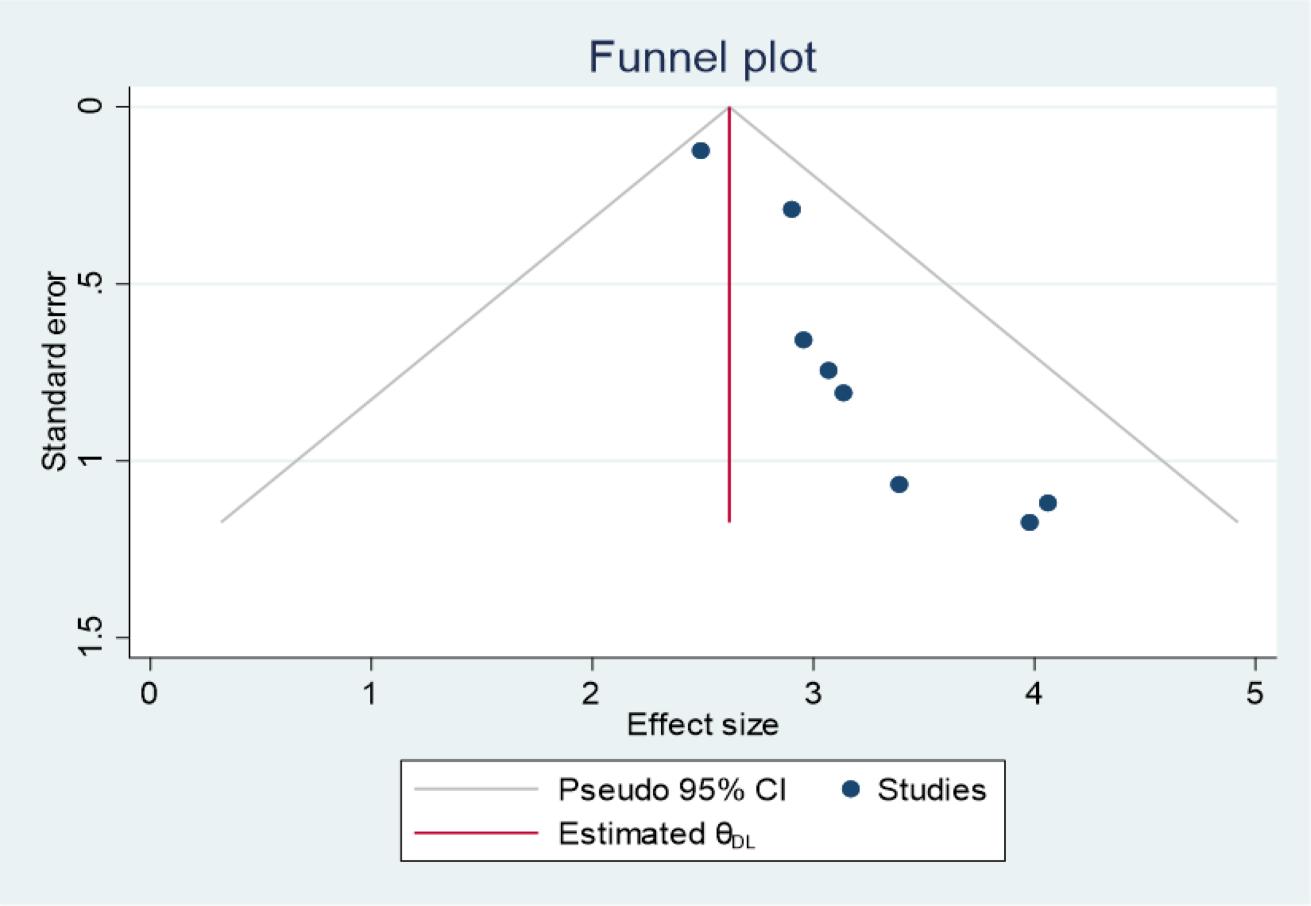
Funnel plot showing publication bias for the magnitude of second-trimester induced abortion studies in Ethiopia, 2024.

**Figure 5:**
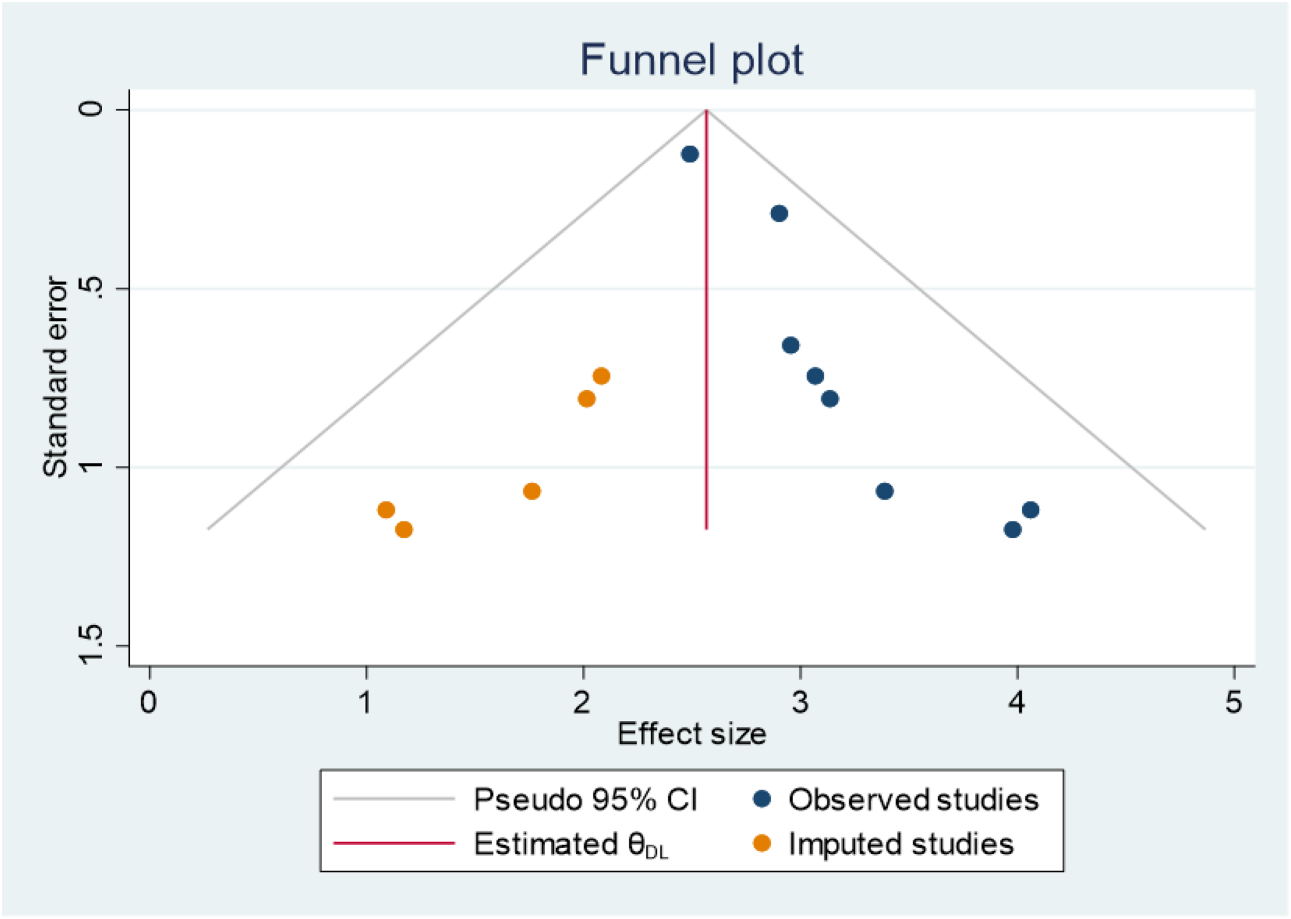
Funnel plots displaying the findings of trim-and-fill analysis for the magnitude of second-trimester induced abortion in Ethiopia, 2024.

The asymmetry of the primary studies included in the funnel plot suggests the presence of publication bias (Figure 4). Additionally, the *p*-value from Egger's regression test (*P* = 0.03) further supports this finding. Therefore, we conducted a trim and fill analysis to address the publication bias (Figure 5).

### Factors associated with second-trimester induced abortion

This systematic review and meta-analysis identified different factors associated with second-trimester induced abortion in Ethiopia. The analysis included variables that were found to have a significant association with second-trimester induced abortion in at least two primary studies. The results showed that delay in confirming pregnancy, irregular nature of menses, rural residence, and unplanned pregnancy were all significantly associated with second-trimester induced abortion.

Five primary studies suggested that delay in confirming pregnancy is significantly associated with second-trimester induced abortion (12, 19, 26, 27, 29). Women who delay confirming their pregnancy are 4.33 times more likely to have a second-trimester induced abortion compared to those who confirm their pregnancy earlier (POR = 4.33, 95% CI: 2.25–8.32). The primary studies showed significant variability (heterogeneity between studies) in their results (*I*² = 86.4%, *p* < 0.0001). Another five primary studies also showed that there was a significant association between the irregular nature of menses and second-trimester induced abortion (12, 27–30).

Women with regular menstrual cycles were 68% less likely to have a second-trimester induced abortion compared to those with irregular menstrual cycles (POR = 0.32, 95% CI: 0.18–0.56). However, the primary studies showed significant heterogeneity (*I*² = 85.3%, *p* < 0.0001), so a random effects model was used (Table 4). Four primary studies (19, 27, 28, 30) found a significant association between residence and second-trimester induced abortion. Women living in urban areas were 62% less likely to have a second-trimester induced abortion compared to those living in rural areas (POR = 0.38, 95% CI: 0.30–0.49). There was no heterogeneity between these studies (*I*² = 0.0%, *p* = 0.551), indicating consistent results across the studies. Similarly, two primary studies (12, 25) demonstrated a significant association between pregnancy status and second-trimester induced abortion. Women with planned pregnancies were 72% less likely to have a second-trimester induced abortion compared to those with unplanned pregnancies (POR = 0.28, 95% CI: 0.18–0.42). These studies also showed no heterogeneity (*I*² = 0.0%, *p* = 0.647), suggesting uniform findings (Table 4).

**Table 4:** Factors associated with the second trimester induced abortion in Ethiopia, 2024.

Variables	Category	Pooled OR 95%CI	P-value	I^2^
Delay to confirm pregnancy (12, 19, 26, 27, 29)	Yes vs. No	4.33 (2.25–8.32)	<0.0001	86.4%
Nature of menses (12, 27–30)	Regular vs. Irregular	0.32 (0.18–0.56)	<0.0001	85.3%
Residence (19, 27, 28, 30)	Urban vs. Rural	0.38 (0.30–0.49)	0.551	0.0%
Pregnancy intention (12, 25)	Planned vs. Unplanned	0.28 (0.18–0.42)	0.647	0.0%

## Discussion

Second-trimester abortions contribute disproportionately to maternal morbidity and mortality as compared to first-trimester abortions, especially in low-resource countries with limited access to safe second-trimester abortions. As far as we know, this study is the first of its kind to determine the national magnitude and factors associated with second-trimester induced abortion in Ethiopia using a systematic review and meta-analysis. The findings of this study will be useful for policymakers, healthcare providers, and researchers working towards reducing the incidence of second-trimester induced abortion in Ethiopia.

The pooled magnitude of second-trimester induced abortion was found to be 29.10% (95% CI: 19.96–38.24). This finding aligns with a multilevel study conducted in Ethiopia (32%) (39). The similar findings may be due to comparable socio-economic and healthcare access conditions, which influence the rates of second-trimester abortions. Women in these regions might face similar barriers to early abortion services, such as limited access to healthcare facilities, lack of awareness, and socio-cultural factors that delay decision-making. These barriers can lead to a higher incidence of second-trimester abortions. Additionally, the results align with studies conducted in India (23%) (40), and South Africa (25%) (41), which further reinforces the reliability and validity of our findings. These comparable prevalence rates suggest that there may be similar underlying factors influencing the occurrence of second-trimester induced abortions across these diverse settings.

However, the findings of our systematic review and meta-analysis indicate a higher prevalence compared to the globally reported prevalence of 10%–15% (42), as well as studies conducted in Mexico (8.10%–13.4%) (43, 44), United Kingdom (10%) (13), and the Netherland (6.6%) (45). The variations in these findings could be attributed to differences in access to abortion services, study periods, sample sizes, educational status, and socio-cultural conditions. Furthermore, differences in sociocultural norms and women's health-seeking behaviors between study areas may also contribute to these discrepancies.

In this review, subgroup analysis by publication year indicated that studies published in 2020 or later had a higher magnitude of second-trimester induced abortion (*P* = 33.37%; CI: 21.60–45.94) compared to those published before 2020 (*P* = 15.46%; CI: 8.47–22.45). The increased availability and accessibility of safe and legal abortion services in the second trimester, along with the growing acceptance of later abortions among doctors and the public, may explain the higher prevalence of second-trimester abortions since 2020. Additionally, this could lead to improved reporting data. This could be attributed to government or non-governmental organizations’ efforts to expand reproductive healthcare facilities and train healthcare providers. Furthermore, newer studies may reflect more current societal trends, healthcare policies, and evolving perceptions surrounding abortion, which could also influence the observed rates.

This study found that a delay in confirming pregnancy was significantly associated with an increased likelihood of second-trimester induced abortions. Women who delayed confirming their pregnancy were 4.33 times more likely to undergo a second-trimester induced abortion compared to those who confirmed their pregnancy earlier (POR = 4.33, 95% CI; 2.25, 8.32). This finding is supported by studies done in UK (46), US (47, 48), China (49), and South Africa (50). A possible reason for the significant association between delaying pregnancy confirmation and the increased likelihood of second-trimester induced abortions is that inadequate access to timely healthcare information and services could cause both delayed pregnancy diagnosis and later abortion. When women delay confirming their pregnancies, they may miss critical windows for accessing early abortion services or other reproductive health care options. This delay could stem from various factors, such as a lack of awareness about early signs of pregnancy, misinformation about reproductive health, or social and cultural stigmas surrounding pregnancy and abortion.

This meta-analysis study revealed that there was a significant association between the nature of menses and second-trimester induced abortion. Women who have regular menstrual cycles are 68% less likely to undergo second-trimester induced abortion than those who have irregular menstrual cycles (POR = 0.32, 95% CI; 0.18–0.56). This finding is supported by studies conducted in the US (47, 48). The possible reason might be menstrual irregularities can make it difficult for women to accurately determine the timing of their last menstrual period, which is used to estimate gestational age. This delay in detecting pregnancy may result in a later diagnosis and subsequent delay in seeking abortion services, leading to an increased likelihood of second-trimester abortion.

Women living in urban areas were significantly less likely to have a second-trimester induced abortion compared to those in rural areas in our study (POR = 0.38, 95% CI: 0.30–0.49). This finding is consistent with studies conducted in Nigeria (51), Canada (52), and a multilevel study in Ethiopia (39). Another systematic review and meta-analysis on induced abortion in Africa found that the prevalence of induced abortion was significantly higher in rural areas compared to urban areas. This study highlighted that women in rural areas often face barriers such as limited access to healthcare services, lack of education, and socio-economic challenges, which contribute to the higher rates of second-trimester abortions (1). The consistency of these findings with the current study underscores the importance of addressing these barriers to reduce the incidence of second-trimester abortions in rural areas. This review found that women with planned pregnancies are significantly less likely to have a second-trimester induced abortion compared to those with unplanned pregnancies (POR = 0.28, 95% CI: 0.18–0.42). This finding is supported by studies conducted in (53), and Burkina Faso (54). Additionally, a systematic review and meta-analysis conducted in Africa corroborates our findings (1). Unplanned pregnancies may lead to more abortions in general, but it is not necessarily the cause of delayed abortions, as the higher overall number of abortions among women with unplanned pregnancies includes both first and second-trimester abortions. According to the World Health Organization, six out of ten unintended pregnancies end in induced abortion (2). Additionally, a report by the United Nations Population Fund highlights that unintended pregnancies have profound consequences for societies, women, and girls, with over 60% of unintended pregnancies ending in abortion (9). The higher likelihood of second-trimester abortions among women with unplanned pregnancies is due to emotional stress, financial constraints, and logistical challenges, which collectively delay decision-making and access to timely abortion services (21). This study emphasizes the critical role of family planning and counseling in reducing the rates of unplanned pregnancies and, consequently, second-trimester abortions.

In Ethiopia, several effective interventions have been implemented to decrease the incidence rates of second-trimester induced abortion. These include widespread and inclusive comprehensive sexual education (CSE) programs that equip individuals with the knowledge to make informed decisions about their sexual health (21, 55). However, CSE is a controversial topic, with varying claims about its effectiveness and appropriateness. While some studies support its positive impact (55), others, such as those by Ericksen and Weed (56) and the Cochrane review (57), highlight the limited evidence that comprehensive sexuality education alone effectively improves sexual and reproductive health outcomes. Additionally, ensuring the availability and affordability of accessible contraceptive services helps prevent unintended pregnancies. Providing supportive healthcare services that are nonjudgmental and compassionate ensures that individuals receive the care they need without stigma. Finally, strengthening policies and advocating for reproductive health rights and healthcare access guarantees that individuals have the necessary legal and societal support to make autonomous decisions about their reproductive health.

This systematic review and meta-analysis have some limitations, including small sample size in certain studies, and the absence of studies from certain regions which may impact the overall generalizability of the findings. Additionally, the presence of heterogeneity across the studies may affect the overall estimate of the magnitude of second-trimester induced abortion.

## Conclusions and recommendations

The magnitude of induced second-trimester abortions in Ethiopia is significantly higher than the global data. This meta-analysis revealed that delaying pregnancy confirmation, having an irregular menstrual cycle, residing in rural areas, and having unplanned pregnancies are associated with an increased likelihood of second-trimester induced abortions. These findings highlight the critical need for targeted interventions to address these factors and reduce the incidence of second-trimester abortions. It is essential to implement targeted interventions aligned with the principles of the International Conference on Population and Development (ICPD) Program of Action from Cairo in 1994 (58). The high prevalence of second-trimester-induced abortions may suggest gaps in the accessibility and availability of early abortion services. This potential gap highlights the need for further investigation into the factors contributing to delayed access to abortion care. Strengthening healthcare infrastructure, particularly in rural and underserved areas, can ensure that women have timely access to abortion care. The top priority should always be to prevent unintended pregnancies and improve women's health by providing essential services on time. Implementing comprehensive reproductive health education programs that provide information about menstrual cycles, family planning options, fertility awareness, and the importance of early pregnancy confirmation can help avoid delays in seeking abortion services. Women should also be informed about the risks associated with second-trimester induced abortion and the importance of seeking timely medical care.

## Data Availability

The raw data supporting the conclusions of this article will be made available by the authors, without undue reservation.
