# Supplementary material for: Epidemiology of second trimester induced abortion in Ethiopia: a systematic review and meta-analysis
Source: Front Glob Womens Health. 2025 Apr 28;6:1452114. doi: 10.3389/fgwh.2025.1452114 (PMC12066765; doi:10.3389/fgwh.2025.1452114)
Supplement: Supplementary file 2 [file Table2.docx]

**Quality assessment of articles using Newcastle - Ottawa quality assessment Scale (NOS): (Adapted for cross-sectional studies)**

|  | **Selection(5*)** | | | | **Comparability(2*)** | **Outcome(3*)** | | **Total score** |
| --- | --- | --- | --- | --- | --- | --- | --- | --- |
| Study ID | Representativeness of the sample(1*) | Sample size(1*) | Non- respondents(1*) | Ascertainment of the exposure(2**) | Confounding factors controlled  (2**) | Assessment of outcome  (2**) | Statistical test(2*) | Total quality score out of 10* |
| Tesfaye B et al [1] | * |  | * | * | ** | ** | * | 8 |
| Mohammed M et al[2] | * | * | * | * | * | ** | * | 8 |
| Abebe M et al[3] | * | * | * | * | ** | ** | * | 9 |
| Kebede K et al[4] | * |  | * | * | * | ** | * | 8 |
| Mulat A et al[5] | * | * | * | * | * | ** | * | 8 |
| Bonnen I et al[6] | * | * |  | * | * | ** | * | 7 |
| Abdi T et al [7] | * | * | * | * | * | ** | * | 8 |
| Dagnaw EH et al [8] | * | * | * | * | * | * | * | 7 |

**Descriptions of quality measurement adapted for cross sectional study**

**Selection: (Maximum 5 stars or 5 points)**

1) Representativeness of the sample:

1. Truly representative of the average in the target population. * (all subjects or random sampling): **1 point**
2. Somewhat representative of the average in the target population. * (nonrandom sampling) : **1 point**
3. Selected group of users: **0**
4. d) No description of the sampling strategy: 0

2) Sample size:

1. Justified and satisfactory: **1 point**
2. Not justified: **0**

3) Non-respondents:

1. Comparability between respondents and non-respondents characteristics is established, and the response rate is satisfactory: **1 point**
2. The response rate is unsatisfactory, or the comparability between respondents and non-respondents is unsatisfactory: **0**
3. No description of the response rate or the characteristics of the responders and the non-responders: **0**

4) Ascertainment of the exposure (risk factor):

1. Validated measurement tool : **(2points)**
2. Non-validated measurement tool, but the tool is available or described: **(1 point)**
3. No description of the measurement tool. **0**

**Comparability: (Maximum 2 stars or 2 points)**

1) The subjects in different outcome groups are comparable, based on the study design or analysis. Confounding factors are controlled.

1. The study controls for the most important factor (select one): 1 point
2. The study control for any additional factor: 1 point

**Outcome: (Maximum 3 stars or points)**

1) Assessment of the outcome:

1. Independent blind assessment: **2 points**
2. Record linkage: **2 points**
3. Self-report: **1 point**
4. No description: **0**

2) Statistical test:

1. The statistical test used to analyse the data is clearly described and appropriate, and the measurement of the association is presented, including confidence intervals and the probability level (p value): **1 point**
2. The statistical test is not appropriate, not described or incomplete. **0**

**Note: 1 asterisk or star (*) is equivalent to 1 point**

**Decisions of on the quality of the studies were based on the sum or total score:**

- **High quality studies: 7-10 points**
- **Low quality studies: 0-6 points**

**Quality of assessment of articles using Newcastle - Ottawa quality assessment Scale (NOS) Adapted for case control studies**

| Studies | **Selection(4*)** | | | | **Comparability(2*)** | **Exposure (4*)** | | | **Total score** | **Study quality** |
| --- | --- | --- | --- | --- | --- | --- | --- | --- | --- | --- |
|  | Is the case definition adequate  (1*) | Representativeness of the Cases (1*) | Selection of Controls  (1*) | Definition of Controls (1*) | Comparability of cases and controls on the basis of the design or analysis (2*) | Ascertainment of exposure  (2*) | Same method of ascertainment for cases and controls  (1*) | Non-Response rate(1*) |  |  |
| **Wasihun Y et al[9]** | * | * | * | * | ** | ** | * | * | 10 | High quality |
| **Addisu E et al[10]** | * | * | * | * | * | ** | * | * | 9 | High quality |

**Descriptions of quality measurement adapted for case control studies**

**Selection: (Maximum 4 stars or 4 points)**

1. **Is the Case Definition Adequate?**
2. Requires some independent validation (e.g. >1 person/record/time/process to extract information, or reference to primary record source such as x-rays or medical/hospital records)
3. Record linkage (e.g. ICD codes in database) or self-report with no reference to primary record
4. No description
5. **Representativeness of the Cases**
6. All eligible cases with outcome of interest over a defined period of time, all cases in a defined catchment area, all cases in a defined hospital or clinic, group of hospitals, health maintenance organisation, or an appropriate sample of those cases (e.g. random sample)
7. Not satisfying requirements in part (a), or not stated.
8. **Selection of Controls**

This item assesses whether the control series used in the study is derived from the same population as the cases and essentially would have been cases had the outcome been present.

1. Community controls (i.e. same community as cases and would be cases if had outcome)
2. Hospital controls, within same community as cases (i.e. not another city) but derived from a hospitalised population
3. No description
4. **Definition of Controls**
5. If cases are first occurrence of outcome, then it must explicitly state that controls have no history of this outcome. If cases have new (not necessarily first) occurrence of outcome, then controls with previous occurrences of outcome of interest should not be excluded.
6. No mention of history of outcome

***COMPARABILITY (maximum 2 stars or points)***

- Either cases or controls must be matched in the design and/or confounders must be adjusted for in the analysis: **
- Statements of no differences between groups or that differences were not statistically significant are not sufficient for establishing comparability.
- Note: If the odds ratio for the exposure of interest is adjusted for the confounders listed, then the groups will be considered to be comparable on each variable used in the adjustment.

***EXPOSURE (maximum 4 stars or points)***

1. Ascertainment of Exposure (maximum 2 points or stars)
2. Non-Response Rate ( 1 star or point)
3. Same method of ascertainment for cases and controls (1 star or point)

**Note: 1 asterisk or star (*) is equivalent to 1 point**

**Decisions of on the quality of the studies were based on the sum or total score:**

- **High quality studies: 7-10 points**
- **Low quality studies: 0-6 points**

1. Tesfaye B, Tewabe M, Ferede A, Dawson A. Induced Second Trimester Abortion and Associated Factors at Debre Markos Referral Hospital: Cross-Sectional Study. Women's Health. 2020;16:1745506520929546.

2. Mohammed M, Wesenu M. Prevalence and determinants associated with second trimester termination of pregnancy in Harari city, Ethiopia; Cross sectional study. 2021.

3. Abebe M, Mersha A, Degefa N, Molla W, Wudneh A. Magnitude of second-trimester-induced abortion and associated factors among women who received abortion service at public hospitals of Arba Minch and Wolayita Sodo towns, southern Ethiopia: A cross-sectional study. Front Glob Womens Health. 2022;3:969310. Epub 20221014. doi: 10.3389/fgwh.2022.969310. PubMed PMID: 36312870; PubMed Central PMCID: PMCPMC9614144.

4. Kebede K, Gashawbeza B, Gebremedhin S, Tolu LB. Magnitude and Determinants of the Late Request for Safe Abortion Care Among Women Seeking Abortion Care at a Tertiary Referral Hospital in Ethiopia: A Cross-Sectional Study. International Journal of Women's Health. 2020;12:1223.

5. Mulat A, Bayu H, Mellie H, Alemu A. Induced second trimester abortion and associated factors in Amhara region referral hospitals. BioMed research international. 2015;2015.

6. Bonnen KI, Tuijje DN, Rasch V. Determinants of first and second trimester induced abortion-results from a cross-sectional study taken place 7 years after abortion law revisions in Ethiopia. BMC pregnancy and childbirth. 2014;14(1):1-9.

7. Abdi T, Assefa F, Debela DT. Factors Associated With Delay in Seeking Abortion Care Until the Second Trimester at Jimma University Medical Centre: A Prospective Cross‐Sectional Study Jimma, Southwest Ethiopia. Reproductive, Female and Child Health. 2024;3(4):e70008.

8. Dagnaw EH, Berta M, Cherkos EA, Mequannent EW. Magnitude of late presentation for induced abortion care services and the associated factors among reproductive-age women at selected hospitals in the South Gondar district of Northwest Ethiopia in 2023: a multicentred, cross-sectional study. BMJ open. 2024;14(11):e081946.

9. Wasihun Y, Mekonnen T, Asrat A, Dagne S, Menber Y, Fentahun N. Determinants of Second-Trimester Safe Termination of Pregnancy in Public Health Facilities of Amhara Region, Northwest Ethiopia: An Unmatched Case-Control Study. Advances in Public Health. 2021;2021.

10. Addisu E, Admassu B, Ayele WM, Angaw K, Adane B. Determinants of induced abortion among second-trimester pregnant women attending Dessie City health facilities, Northeast Ethiopia: a case-control study. HIV & AIDS Review.
